# The mitochondrial genome of *Medetera* sp. (Diptera: Dolichopodidae)

**DOI:** 10.1080/23802359.2019.1696246

**Published:** 2019-12-09

**Authors:** Qilemoge, Chen Lin, Noor Fatima, Ding Yang

**Affiliations:** a College of Plant Protection, China Agricultural University, Beijing, China;; b Institute of Life Science and Technology, Inner Mongolia Normal University, Huhhot, China

**Keywords:** Mitochondrial genome, Medeterinae, phylogenetics

## Abstract

The long-legged fly *Medetera* sp. (Genbank accession number: MN604695) belongs to the subfamily Medeterinae of Dolichopodidae. The mitogenome of *Medetera* sp. was sequenced, the first representative of the mitogenome of the subfamily. The mitogenome is 14,740 bp totally, consisting of 13 protein-coding genes, two rRNAs, and 22 transfer RNAs. All genes have similar locations and strands with that of other published species of Dolichopodidae. The nucleotide composition biases toward A and T, which together made up 67.6％ of the entirety. Bayesian inference analysis strongly supported the monophyly of Dolichopodidae. It suggested that subfamily Medeterinae is the sister group of subfamily Rhaphiinae.

Medeterinae belongs to the family Dolichopodidae, and the *Medetera* Fischer von Waldheim, 1819 is a larger genus of Medeterinae with 355 extant species and seven fossil species known from the world (Yang et al. 2006; Yang et al. 2011; Grichanov 2017).

The adult specimens of *Medetera* sp. (Genbank accession number: MN604695) used for this study were collected from Wang Lang Nature Reserve (32°91′52″N, 104°16′38″E, 2403 m) of Sichuan Province in China in 2016. The specimens of *Medetera* sp. were deposited in the Entomological Museum (Accession Number: D-M-1) of China Agricultural University (CAU). The total genomic DNA was extracted from the whole body (except head) of the specimen using the QIAamp DNA Blood Mini Kit (Qiagen, Germany) and stored at −20 °C until needed. The nearly complete mitogenome of *Medetera* sp. is 14,740 bp. It encoded 13 PCGs, 22 tRNA genes, two rRNA genes and the control region could not be sequenced entirely in this study and were similar with related reports before (Kang et al. 2016; Li et al. 2016; Wang, Ding, et al. 2016; Wang, Li, et al. 2016; Wang, Liu, et al. 2016; Wang, Wang, et al. 2016; Li et al. 2017; Zhou et al. 2017; Qilemoge, Gao, et al. 2018; Qilemoge, Zhang, et al. 2018; Hou et al. 2019; Qilemoge et al. 2019). The nucleotide composition of the mitogenome was biased toward A and T, with 67.6% of A + T content (A = 37.8%, T = 29.9%, C = 20.8%, and G = 11.6%). The A + T content of PCGs, tRNAs, and rRNAs is 65.3%, 72.8%, and 76.2%, respectively. The total length of all 13 PCGs of *Medetera* sp. is 11,213 bp. All PCGs in *Medetera* sp. utilize the conventional translational start codons for invertebrate mtDNA. For example, six PCGs (*COII*, *COIII*, *ATP6*, *ND4*, *ND4L*, and *CYTB*) initiated with ATG codons, two PCGs (*ND3* and *ND5*) initiated ATT codons, three PCGs (*ND2*, *ND1*, and *ND6*) initiated ATA codons, *ATP8* initiated ATC codon, and *COI* initiated TCG codon. Eight PCGs used the typical termination codons TAA and three PCGs (*ND1*, *ND3*, and *CYTB*) used TAG in *Medetera* sp.

Phylogenetic analysis was performed based on the nucleotide sequences of 13 PCGs and two rRNAs from 15 Diptera species. Bayesian (BI) analysis (Figure 1) showed that monophyletic Empidoidea was assigned to be the sister group to the clade of Xylophagidae and Asilidae. For the phylogeny of Empidoidea, monophyletic Empididae was assigned to the sister to monophyletic Dolichopodidae. For the phylogeny of Dolichopodidae, Medeterinae was assigned to the sister of Rhaphiinae. The phylogenetic relationship within Empidoidea is very clear: Empididae + (Hydrophorinae + Sympycninae) + (Dolichopodinae + (Sciapodinae + (Diaphorinae + (Rhaphiinae + Medeterinae))))). The position of Medeterinae was also supported by the morphological study (Yang et al. 2011). The mitogenome of *Medetera* sp. could provide important information for the further studies of Dolichopodidae phylogeny.

**Figure 1.**
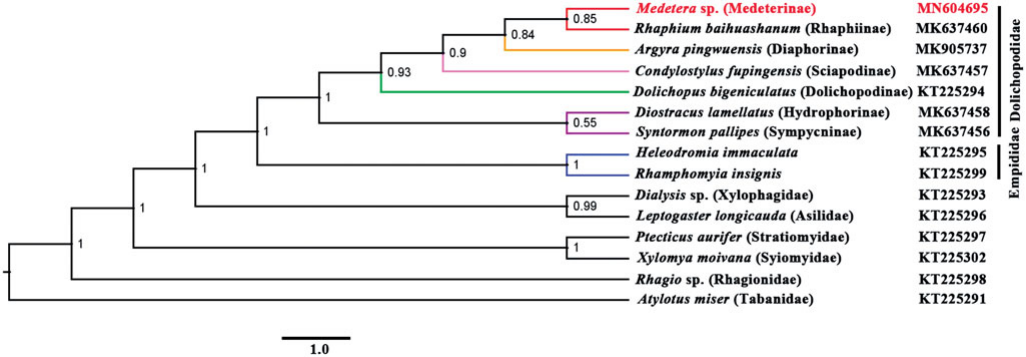
The phylogenetic tree of Bayesian interface analysis based on 13 PCGs and two rRNAs from 15 species.
